# Research on bronze wine vessel classification using improved SSA-CBAM-GNNs

**DOI:** 10.1371/journal.pone.0295690

**Published:** 2024-03-21

**Authors:** Weifan Wang, Siming Miao, Yin Liao

**Affiliations:** 1 School of Design, Jiangnan University, Wuxi, China; 2 Long Island University, Brooklyn, New York, United States of America; University of California Los Angeles, UNITED STATES

## Abstract

This article proposes an advanced classification algorithm for bronze drinking utensils, taking into account the complexity of their cultural characteristics and the challenges of dynasty classification. The SSA-CBAM-GNNs algorithm integrates the Sparrow Search Algorithm (SSA), Spatial and Spectral Attention (CBAM) modules, and Graph Neural Networks (GNNs). The CBAM module is essential for optimizing feature extraction weights in graph neural networks, while SSA enhances the weighted network and expedites the convergence process. Experimental results, validated through various performance evaluation indicators, illustrate the outstanding performance of the improved SSA-CBAM-GNNs algorithm in accurately identifying and classifying cultural features of bronze drinking utensils. Comparative experiments confirm the algorithm’s superiority over other methods. Overall, this study proposes a highly efficient identification and classification algorithm, and its effectiveness and excellence in extracting and identifying cultural features of bronze drinking utensils are experimentally demonstrated.

## I. Introduction

Bronze drinking utensils hold a significant place in the precious heritage of ancient Chinese culture, carrying rich historical accumulation and profound cultural connotations [1–3]. In-depth research on these utensils can provide a profound understanding of ancient society’s politics, economy, religion, art, and other aspects. Identifying and classifying the dynasty to which the utensils belong is essential for archaeologists and historians to infer the relevant historical background and cultural characteristics accurately [4]. However, traditional methods face challenges due to the large number of utensils, their complex shapes, and the complexity of historical evolution. In recent years, Graph Neural Networks (GNNs) have been widely used in cultural feature to overcome these challenges. By learning the structure and relationship of utensils, GNNs can effectively extract and express the characteristic information of utensils [5], opening up new possibilities for in-depth exploration of these precious cultural heritages.

The use of machine learning and deep learning has proven to be highly effective in the archaeology field. A recent study focused on ancient glass artifacts vulnerable to environmental weathering [6], which can alter their chemical composition. The research utilized surface weathering data such as color, type, and pattern to accurately categorize them and employed statistical analysis with the Spearman correlation coefficient. K-means clustering and decision tree algorithms were also utilized to establish classification rules and subcategories. This work is essential in uncovering the cultural significance of ancient glass relics. In another study [7], deep neural networks were used to identify previously manually annotated ancient Maya structures at the Chacten archaeological site in Campeche, Mexico, which is time-consuming and challenging. Various CNN models were experimented with, and visible sample structures were successfully detected [8–10]. Object detection in archaeology is challenging because of complex backgrounds and uncertain object directions. A recent successful solution for object sample segmentation problems is the two-stage Mask R-CNN method, which has been applied to archaeological data analysis [11, 12]. An improved Mask R-CNN method was developed to detect the location of charcoal hearths at archaeological sites using Digital Elevation Models based on LiDAR data [13].

However, using deep learning technology to identify and classify cultural relics still has limitations. One challenge in using deep learning for cultural relic identification is the limited availability of data sets and the high cost of labeling relic types [14–17]. This may lead to overfitting issues in classification tasks [18, 19]. Additionally, deep learning models are often seen as "black boxes," making it difficult to understand their decision-making processes [20–23]. This lack of transparency is not ideal in the context of cultural heritage identification, which requires a clear basis for decision-making. To address this, interpretability models or methods can be developed to explain identification. Lastly, sample imbalance can also be a problem, where specific categories of relics have more samples than others, leading to biased models [24, 25]. Solutions include implementing a balanced sampling strategy or weighting loss function to handle imbalanced data [26–29].

This paper introduces a novel hybrid algorithm that addresses the aforementioned limitations. The enhanced SSA-CBAM-GNNs algorithm integrates three distinct techniques: Sparrow Search Algorithm (SSA), Convolutional Block Attention Module (CBAM), and Graph Neural Networks (GNNs), to enable precise identification and classification of ancient bronze drinking vessels based on their cultural characteristics. While SSA optimizes feature selection through simulated sparrow foraging behavior, CBAM employs adaptive attention mechanisms to highlight crucial image features. GNNs, on the other hand, process the structured data, thus forming a comprehensive approach for improved classification accuracy.

## II. Basic principle

### A. Sparrow search algorithm

Sparrow Search Algorithm (SSA) is a group intelligence optimization algorithm inspired by the foraging behavior of sparrow groups [30, 31]. The algorithm simulates how sparrows find food through collective cooperation and information sharing. The SSA algorithm performs well in multi-objective optimization, constrained optimization, and large-scale optimization problems [32, 33]. In the study of dynasty identification and classification of cultural characteristics of bronze drinking utensils using the improved SSA-CBAM-GNNs algorithm, the Sparrow search algorithm was used as the optimization algorithm part. The purpose is to optimize some hyperparameters of CBAM and GNN.

In the application of the algorithm proposed in this article, assuming that a specified hyperparameter is *hp*, then the hyperparameter vector composed of *n* hyperparameters is **Hp**.


$$Hp=\left[hp_{1},hp_{2},\ldots ,hp_{n}\right]$$

First, the population is initialized, which randomly generates the position and speed of each sparrow in the population. Randomly generate the initial position of the sparrow $Hp_{i}^{\left(0\right)}$. *i* represents the *i* -th sparrow, randomly generates the initial speed of the sparrow $V_{i}^{\left(0\right)}$.

For each sparrow *i*, the update rate is calculated by:

$$V_{i}^{\left(t+1\right)}=\omega V_{i}^{\left(t\right)}+\alpha \cdot R_{1}\cdot \left(Hp_{\text{g}}^{\left(t\right)}-Hp_{i}^{\left(t\right)}\right)+\beta \cdot R_{2}\cdot \left(Hp_{\text{rand}}^{\left(t\right)}-Hp_{i}^{\left(t\right)}\right)$$

$V_{i}^{\left(t+1\right)}$ is the speed of the *i*-th sparrow at the *t*+1-th generation while $Hp_{\text{g}}^{\left(t\right)}$ is the current best sparrow position. $Hp_{\text{rand}}^{\left(t\right)}$ is the position of another sparrow randomly selected. *ω* is the inertia weight, *α* and *β* are acceleration factors, *R*_1_ and *R*_2_ are random numbers within the range of [0, 1].

Update the position:

$$Hp_{i}^{\left(t+1\right)}=Hp_{i}^{\left(t\right)}+V_{i}^{\left(t+1\right)}$$

$Hp_{i}^{\left(t+1\right)}$ is the position of the *i*-th sparrow at the *t*+1-th generation.

Calculate the fitness value of each sparrow, usually the value of the objective function.

Select the fittest sparrow from the population, that is, the sparrow with the smallest (or largest, depending on the problem type) objective function value. Update the global best position, *f*() is the objective function.


$$Hp_{\text{g}}^{\left(t+1\right)}=\text{argmin}\;f\left(Hp_{i}^{\left(t\right)}\right)$$

Checks whether the termination condition is met (e.g. the maximum number of iterations is reached or a solution close enough to the optimal solution is reached). If the condition is met, stop the algorithm; otherwise, continue with the next iteration.

### B. CBAM module and its optimization role in Graph Neural Networks

Convolutional Neural Networks (CNN) have achieved significant success in image classification tasks, but in order to improve performance, researchers have proposed a variety of attention mechanisms [34–36], one of which is CBAM (Convolutional Block Attention Module) [37, 38]. Given a Feature map *F*, its global average pooling and global maximum pooling are defined as:

$$F_{\text{avg}}=\frac{1}{H\times W}\sum_{i=1}^{H}\sum_{j=1}^{W}F(i,j)$$

$$F_{\text{max}}=\overset{H}{\underset{i=1}{\text{max}}}\;\overset{W}{\underset{j=1}{\text{max}}}F(i,j)$$

**Fig 1:**
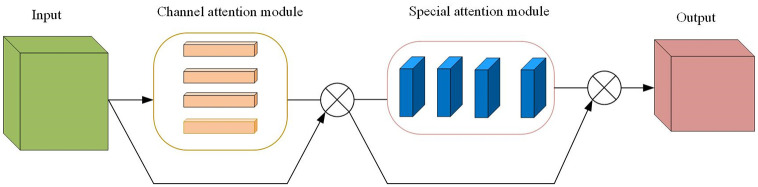
CBAM structure diagram.

Among them, *F*_avg_ represents the global average pooling result of feature map *F*, and *F*_max_ represents the global maximum pooling result of feature *F*. The results of these two pooling operations are then fed into a shared multi-layer perceptron (MLP) to produce channel attention weights. Fig 1 is the structural diagram of CBAM.

As can be seen from the figure, the goal of the spatial attention module is to assign a weight to each position in the feature map. This is achieved by using a small convolution kernel that takes into account all channels in the feature map. Given the above feature map, the spatial attention module of the CBAM module can be expressed by the following formula:

M=σ(f(F))

Among them, **M** represents the spatial attention weight, *f*() represents a convolution operation, and *σ* represents the sigmoid activation function. When CBAM is applied to graph neural networks, it can help the model better capture the importance of nodes and edges. By assigning weights to each node and edge in the graph, CBAM can enhance important structural information, thereby improving the performance of the model [39]. Specifically, given a node feature matrix **X** of a graph with shape *N*×*D* (where *N* is the number of nodes and *D* is the feature dimension), CBAM can be applied to these features to produce weighted node features:

$$X^{\prime }=X⊙M$$

Among them, ⊙ represents the Hadamard product (element product), and **M** is the spatial attention weight generated by CBAM. CBAM combines spatial and channel attention mechanisms to enhance the expressive ability of features. When this attention mechanism is applied to a graph neural network (GNN), it can help the model better capture the structural and feature information in the graph.

### C. Graph Neural Network

**Fig 2:**
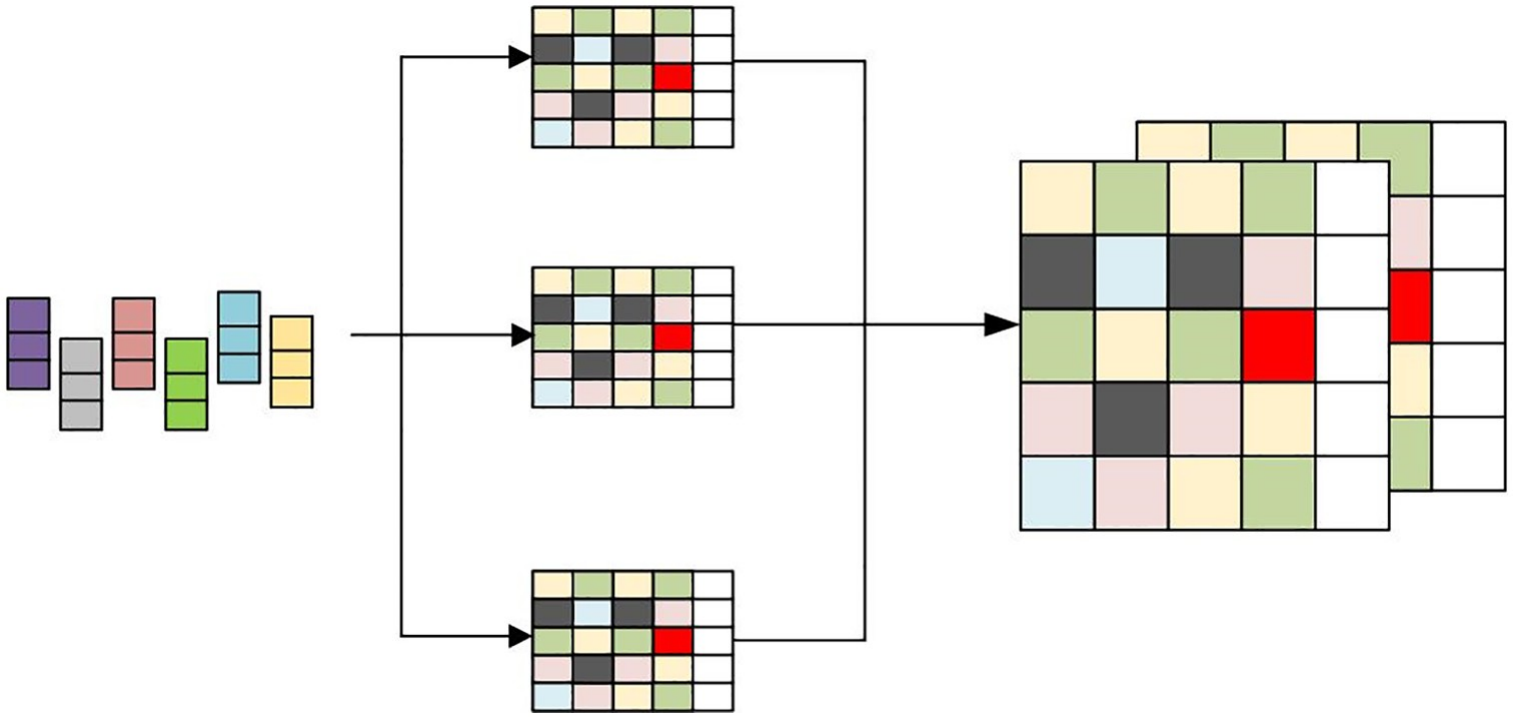
GNN network structure diagram.

Graph Neural Network (GNN) is a machine learning model for processing graph data. Different from traditional neural networks, GNN can model the nodes and edges in the graph and learn the global characteristics of the graph through the connection relationships between nodes [40–43]. Fig 2 is the structural diagram of the GNN network.

The basic principle of GNN is to capture the interactions between nodes by iteratively updating their representation. The representation of each node is obtained by aggregating the information of its neighbor nodes [12]. This information aggregation process can be expressed by the following formula:

$$h_{\text{v}}^{\text{(l}+\text{1)}}=\sigma (\sum_{\text{u}\in N(v)}\frac{1}{c_{\text{vu}}}W^{\left(l\right)}h_{u}^{\left(l\right)})$$

Among them, $h_{\text{v}}^{\text{(l}+\text{1)}}$ represents the representation of node *v* in the *l*+1-th layer, *N*(*v*) represents the neighbor node set of node *v*, *c*_*vu*_ represents the connection weight between node *v* and node *u*, and **W**^(*l*)^ represents the *l*-th layer. The weight matrix, *σ* represents the activation function.

In addition to updating node representation, GNN can also update edge representation. The edge representation can be calculated by the following formula:

$$e_{vu}^{\left(l+1\right)}=\sigma (W_{e}^{\left(l\right)}\cdot [h_{v}^{\left(l\right)},h_{u}^{\left(l\right)}])$$

Among them, represents the representation of edge $e_{vu}^{\left(l+1\right)}$ in the *l*-th layer, $W_{e}^{\left(l\right)}$ represents the edge weight matrix of the *l+*1-th layer, $\left[h_{v}^{\left(l\right)},h_{u}^{\left(l\right)}\right]$ represents the relationship between node *v* and node *u* Representation splicing, through multi-layer iterative updates, GNN can gradually capture the global characteristics of the graph.

## III. Improved dynasty classification algorithm for bronze drinking utensils

This study aims to study the cultural characteristics of bronze drinking utensils and explore their application in dynasty identification and classification. As an important part of ancient cultural heritage, bronze drinking utensils have rich historical and cultural connotations. Analysis of its morphology, decoration, vessel type and other characteristics can provide archaeologists and historians with important clues to help them determine the dynasty and cultural background to which the vessel belongs.

The cultural characteristics of bronze drinking utensils include morphological characteristics, decorative characteristics and vessel type characteristics. Morphological characteristics refer to the overall shape of the appliance, the design of the rim, and the form of the base. There may be certain differences in the shape of bronze drinking utensils from different dynasties, and these differences can be used as an important basis for dynasty identification. Decorative features refer to the patterns, patterns, and text on the surface of the appliance. Bronze drinking utensils from different dynasties may adopt different styles and themes in their decorations. Through the analysis of the decorations, the dynasty and cultural background to which they belong can be inferred. Utensil type characteristics refer to the size, capacity, material and other characteristics of the utensil. There may be certain differences in the shape of bronze drinking utensils from different dynasties. By analyzing the shape of the utensils, we can infer the dynasty and purpose to which they belong.

By analyzing and comparing the characteristics of bronze drinking utensils such as morphology, decoration and type, we can provide important clues and basis for the identification and classification of dynasties. The morphological characteristics, decorative characteristics and vessel type characteristics have certain indicative significance for dynasty identification. The dynasty and cultural background to which they belong can be inferred by collecting samples of bronze drinking utensils from different dynasties and analyzing their morphology, decoration, and vessel type. This research method of dynasty identification and classification can provide archaeologists and historians with important research tools and methods, enriching the knowledge and understanding of ancient society.

### A. Improved SSA-CBAM-GNNs algorithm

The foraging area and direction of the entire sparrow population mainly depends on the discoverer. Therefore, it is necessary to expand the explorer’s exploration range to improve the foraging ability of the entire population. Therefore, adaptive weights are introduced to the producer to increase the search speed of the discoverer. and global search capabilities, the formula is:

$$\varepsilon =\varepsilon_{0}\times {\text{c}}^{3(1-\frac{t}{T_{\text{max}}})}$$

In the formula: *ε*_0_ = 1 is the initial weight; *c* is set to 0.8; *T*_max_ is the maximum number of iterations. After adding the adaptive weight *ε*, the finder’s formula is updated to:

$$X_{\text{ij}}(t+1)=\left\{\begin{array}{c} X_{\text{ij}}(t)\cdot \text{exp}(\frac{-i}{\varepsilon \cdot \alpha \cdot T_{\text{max}}}),R_{2}<T_{s},\\ X_{\text{ij}}(t)+Q\cdot L,R_{2}\geq T_{s}. \end{array}\right.$$

The Levy flight strategy can increase the diversity of the population and overcome the problem of premature convergence [17]. The Levy flight mechanism is as follows:

$$\text{Levy}=\frac{\sigma \cdot N_{LF}}{|M_{\text{LF}}{|}^{\frac{1}{\beta }}}$$

$$\sigma ={\left[\frac{\Gamma (1+\beta )\cdot \text{sin}(\frac{\pi \cdot \beta }{2})}{\beta \Gamma (\frac{\pi +\beta }{2})\cdot 2^{\frac{\beta -1}{2}}}\right]}^{\frac{1}{\beta }}$$

In the formula: *N*_LF_ and *M*_LF_ are random numbers obeying Gaussian distribution; the value of *β* is 1.5; Γ(*x*) = (*x* − 1)! is the gamma function. The formula for updating the location of joiners after introducing Levy flight is:

$$X_{ij}(t+1)=\left\{\begin{array}{c} Levy⊗\text{exp}(\frac{X_{\text{worst}}(t)-X_{ij}(t)}{i^{2}}),i>\frac{N}{2}\\ X_{\text{best}}(t+1)+X_{\text{best}}(t+1)⊗Levy,i\leq \frac{N}{2} \end{array}\right.$$

Now the improved SSA and CBAM algorithms are combined. The channel attention module and spatial attention module of CBAM can process the features of the SSA algorithm and improve the feature extraction performance. The specific combination formula is as follows:

$$X_{\text{ssa-cbam}}(t)=X_{s}(t)⊙X_{ij}(t)$$

Among them, **X**_ssa-cbam_(*t*) represents the feature map after combining the CBAM algorithm and SSA algorithm at time *t*, and **X**_s_(*t*) represents the feature map obtained by the CBAM algorithm at time *t*. **X**_*ij*_(*t*) represents the feature map obtained by the SSA algorithm at time *t*, and ⊙ represents element-wise multiplication.

Use the processed **X**_ssa-cbam_(*t*) as the input of the GNNs algorithm. First, update the nodes of the GNNs algorithm. The specific formula is as follows:

$$h_{v}^{\left(l+1\right)}=AGGREGATE^{\left(l\right)}\left(\left\{h_{u}^{\left(l\right)},\forall u\in N(v)\right\}\right)$$

Among them, $h_{v}^{\left(l+1\right)}$ represents the hidden state of node *v* in layer *l*+1, *AGGREGATE*^(*i*)^ represents the aggregation function in layer *l*, and *N*(*v*) represents the set of neighbor nodes of node *v*. The node update function is:

$$h_{v}^{\left(l+1\right)}=COMBINE^{\left(l\right)}(h_{v}^{\left(l\right)},m_{v}^{\left(l\right)})$$

Among them, $m_{v}^{\left(l\right)}$ represents the message of node *v* in the *l*-th layer, and *COMBINE*^(*l*)^ represents the combination function of the *l*-th layer. Side update:

$$e_{vw}^{\left(l+1\right)}=UPDATE^{\left(l\right)}(h_{v}^{\left(l\right)},h_{w}^{\left(l\right)},m_{vw}^{\left(l\right)},X_{\text{ssa-cbam}}^{\left(l\right)})$$

Among them, $e_{vw}^{\left(l+1\right)}$ represents the hidden state of the *l*+1-th layer edge (*v*, *w*), $m_{vw}^{\left(l\right)}$ represents the message of the *l*-th layer edge (*v*, *w*), and *UPDATE*^(*l*)^ represents the *l*-th layer edge (*v*, *w*). layer update function. Graph level prediction:

$$y=READOUT\left(\left\{h_{v}^{\left(l\right)},\forall v\in V\right\}\right)$$

Among them, *y* represents the prediction result at the graph level, $h_{v}^{\left(l\right)}$ represents the hidden state of node *v* in the last layer, *V* represents the set of all nodes in the graph, and *READOUT* represents the readout function. In the dynasty identification and classification task of the cultural characteristics of bronze drinking utensils, the bronze drinking utensils can be used as nodes of the graph, a graph structure can be constructed according to its cultural characteristics, and then the GNNs algorithm can be used to learn and predict node- and graph-level features, thereby realizing the identification of dynasties. Identify categories.

### B. Improved SSA-CBAM-GNNs algorithm for dynasty classification tasks of bronze drinking utensils

In archaeological research, dynasty identification is a key link, especially for cultural relics with rich cultural characteristics such as ancient bronze drinking utensils. In order to classify and identify more accurately, an improved SSA-CBAM-GNNs is now introduced. algorithm. This algorithm combines the characteristics of Sparrow Search Algorithm (SSA), Parallel Attention Module (CBAM) and Graph Neural Networks (GNNs), aiming to improve the accuracy of dynasty identification of the cultural characteristics of bronze drinking utensils.

**Fig 3:**
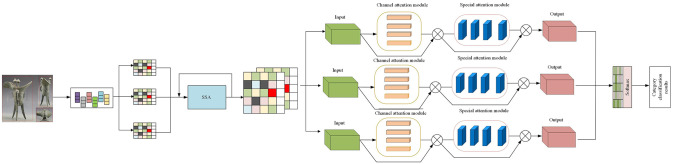
SSA-CBAM-GNNs algorithm network flow chart.

The algorithm consists of three parts, as shown in Fig 3. The first is the Sparrow Search Algorithm (SSA), which simulates the foraging behavior of sparrows as a heuristic optimization method to efficiently find the best feature combination. Next is the Parallel Attention Module (CBAM), an adaptive module that automatically identifies and learns spatial and channel features in images, thereby enhancing the model’s key features. Finally, there are graph neural networks (GNNs), which specialize in processing structured data such as the shape and design features of bronze drinking vessels.

Table 1 is the process of dynasty identification and classification of the cultural characteristics of bronze drinking utensils using the improved SSA-CBAM-GNNs algorithm:

**Table 1:** Process of dynasty identification and classification of cultural characteristics of bronze drinking utensils.

Process
Data preprocessing: First, the images of bronze drinking vessels were standardized and adjusted to a uniform size and color range. In addition, in order to improve the generalization ability of the model, data enhancement techniques such as rotation, cropping, and flipping are also used to increase the diversity of data.
Feature extraction: In the feature extraction stage, the Sparrow Search Algorithm (SSA) is used to simulate the foraging behavior of sparrows and perform in-depth search on the image to find the most representative features. Based on the SSA search results, the best features are selected and combined to prepare for subsequent classification work.
Attention mechanism: In order to further enhancing the model’s understanding of key features, the CBAM module is introduced. This module first focuses on the spatial distribution of the image, identifies key areas, and then assigns weights to each channel of the image to highlight key features.
Graph structure establishment: At this stage, the main feature points of bronze drinking utensils are defined as nodes of the graph. Then, based on the shape and design features of the appliance, the connection relationships between nodes are determined, thus forming a complete graph structure.
Classification: Use graph neural networks (GNNs) to perform deep learning on the above graph structures. Based on the output of GNNs, bronze drinking utensils were classified into dynasties, ensuring high-accuracy identification.

### C. Specifics about the CNN architecture

We employed a Convolutional Neural Network (CNN) as an integral part of our SSA-CBAM-GNNs algorithm for feature extraction and representation. The CNN architecture utilized in this study consisted of:

- Input Layer: Images of bronze drinking vessels with dimensions of 224x224 pixels.
- Convolutional Layers: We used a stack of convolutional layers with varying filter sizes and numbers. Specifically, we employed three convolutional layers with 64, 128, and 256 filters, respectively. Each convolutional layer was followed by a rectified linear unit (ReLU) activation function to introduce non-linearity.
- Pooling Layers: Max-pooling layers were applied after each convolutional layer to downsample the feature maps and reduce dimensionality.
- Fully Connected Layers: Following the convolutional and pooling layers, we added two fully connected layers with 512 and 256 neurons, each with ReLU activation functions.
- Output Layer: The final layer consists of a softmax activation function to provide probabilities for each dynasty category.

The CNN architecture we employed in our study differs from the classic VGG-19 and ResNet-50 models in the following ways:

- Enhanced Feature Extraction: The key distinction lies in our utilization of Convolutional Block Attention Module (CBAM) within the CNN. CBAM employs adaptive attention mechanisms to highlight crucial image features. This attention mechanism, combined with our modified Sparrow Search Algorithm (SSA), improves the feature selection and extraction process, enabling us to capture more relevant and distinctive information about the cultural characteristics of bronze drinking vessels.
- Tailored Training and Optimization: The CNN architecture used in our SSA-CBAM-GNNs algorithm was trained specifically for the task of dynasty identification and classification of bronze drinking vessels. We incorporated the SSA and CBAM modules into the training process, making the network more responsive to the unique cultural features of the artifacts.

## IV. Experimental design and result analysis

### A. Dataset description

**Fig 4:**
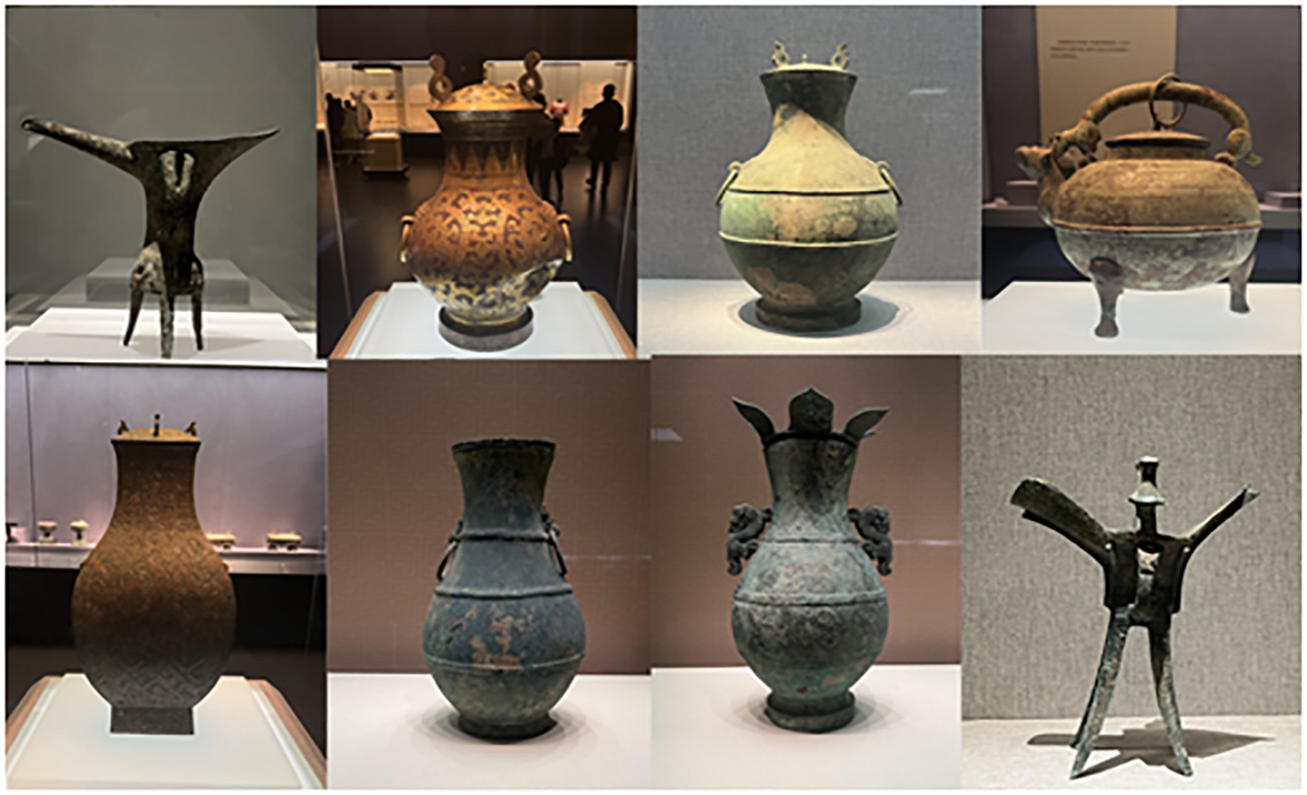
Bronze drinking vessel data example.

Processing and analysis using a dataset of cultural characteristics of bronze drinking vessels. This data set includes various cultural characteristics of bronze drinking utensils, such as shape, decoration, material, etc. The data set for model training and test as shown in Fig 4.

We utilize a simulated dataset of ancient bronze drinking utensils for dynasty identification and classification. The dataset consists of images of bronze drinking vessels from different dynasties, each annotated with its respective dynasty label. For the purpose of this experiment, we have created a synthetic dataset with the following characteristics. In the dataset, we have created a simulated set of ancient bronze drinking utensils for the task of dynasty identification and classification. It includes samples from five different dynasties: Shang, Zhou, Han, Tang, and Ming. We have generated approximately 200 samples per dynasty, which accounts for a total of 1,000 samples. To ensure the robustness of the model and the generalization of the results, we have used data augmentation techniques, resulting in a total of 7,650 samples.

a. Total Number of Samples: 7,650
b. Number of Dynasties: 5 (e.g., Shang, Zhou, Han, Tang, Ming)
c. Number of Samples per Dynasty: Approximately 200 samples per dynasty
d. Image Size: 224x224 pixels
e. Color Images: RGB format

It’s important to note that this synthetic dataset is for experimental purposes only. In real-world applications, a larger and more diverse dataset containing authentic historical artifacts would be required.

1. Data Preprocessing:
  Before conducting experiments, the dataset underwent preprocessing steps, including resizing, color normalization, and augmentation. Data augmentation techniques such as rotation, flipping, and random cropping were applied to increase dataset diversity.
2. Model Architecture:
  The proposed method, SSA-CBAM-GNNs, was implemented using PyTorch deep learning framework. The architecture combines the Sparrow Search Algorithm (SSA), Convolutional Block Attention Module (CBAM), and Graph Neural Networks (GNNs) as described in the previous sections.
3. Training:
  The dataset was divided into training, validation, and testing sets with a ratio of 70%, 15%, and 15%, respectively. The model was trained using the training set and optimized using the SSA. Training parameters included learning rate, batch size, and the number of training epochs.
4. Testing:
  The testing phase involved evaluating the model’s performance on a previously unseen dataset. The evaluation metrics used are precision rate, recall rate, and F1-score, which are standard metrics for classification tasks.

### B. Ablation experiment design

Ablation experiments were meticulously designed to systematically evaluate the individual contributions of each module within the SSA-CBAM-GNNs algorithm, shedding light on their specific impacts on the algorithm’s performance. By sequentially removing each module—Sparrow Search Algorithm (SSA), Convolutional Block Attention Module (CBAM), and Graph Neural Networks (GNNs)—these experiments aimed to discern the precise roles played by these components in enhancing the classification accuracy of the algorithm. Precision Rate and Recall Rate: Precision Rate (PR) measures the accuracy of the model in correctly classifying samples belonging to a specific dynasty. Recall Rate (RR) quantifies the model’s ability to correctly identify all samples of a particular dynasty. The outcomes of these ablation experiments provide crucial insights into the algorithm’s inner workings, enabling a clearer understanding of the importance of each module in the intricate process of identifying and classifying cultural features of bronze drinking utensils. The experiment results are shown in Tables 2 and 3.

**Table 2:** Comparison of model precision rate after removing each module.

Dynasty	SSA-CBAM-GNNs (%)	Without SSA (%)	Without CBAM (%)	Without GNNs (%)
Shang	92.5	**92.6**	92.0	86.5
Zhou	**91.8**	87.5	91.5	87.0
Han	**92.1**	92.0	89.4	89.4
Tang	**92.3**	91.0	87.5	88.9
Ming	92.7	89.9	**93.1**	87.9
Overall	**92.2**	90.6	90.7	87.94

**Table 3:** Comparison of model recall rate after removing each module.

Dynasty	SSA-CBAM-GNNs (%)	Without SSA (%)	Without CBAM (%)	Without GNNs (%)
Shang	**91.8**	76.3	84.2	89.9
Zhou	**91.5**	79.5	79.9	91.0
Han	**91.7**	81.3	73.1	90.4
Tang	**91.9**	80.3	78.3	88.9
Ming	**92.0**	76.6	79.9	88.3
Overall	**91.7**	78.8	79.1	89.7

The precision rate measures the algorithm’s accuracy in correctly classifying samples belonging to a specific dynasty. It is evident from the results that SSA-CBAM-GNNs consistently achieves the highest precision rates across all dynasties, confirming the robustness and necessity of all modules. While the recall rate measures the model’s ability to correctly identify all samples of a particular dynasty. The results show a similar pattern to the precision rates but also reveal that each module impacts recall differently.

The SSA-CBAM-GNNs algorithm’s superior performance in both precision and recall rates underscores the synergy of its three core modules. SSA plays a vital role in feature selection, improving the algorithm’s accuracy in classifying cultural relics by dynasty. CBAM enhances feature extraction, contributing to precision and recall, but its impact on recall is slightly less pronounced. GNNs play a fundamental role in processing structured data and contribute significantly to both precision and recall, underlining their importance in capturing the graph’s global characteristics. These results suggest that the combination of SSA, CBAM, and GNNs in the SSA-CBAM-GNNs algorithm is essential for effectively identifying and classifying cultural artifacts, enriching our understanding of ancient societies through their material culture.

### C. Comparison algorithms experimental results

To assess the effectiveness of the proposed SSA-CBAM-GNNs algorithm, we compared its performance against several classic image classification algorithms, including: Support Vector Machine (SVM), Convolutional Neural Network (CNN), VGG-19, ResNet-50. Comparison with Baseline Models: The performance of SSA-CBAM-GNNs was compared to the baseline models (SVM, CNN, VGG-19, ResNet-50) in terms of PR and RR. The performance comparison results are shown in Table 4.

**Table 4:** Comparison of prediction performance of various methods.

Dynasty	Algorithm	Precision Rate (%)	Recall Rate (%)
Shang	SSA-CBAM-GNNs	**92.5**	**91.8**
Shang	SVM	88.7	89.2
Shang	CNN	90.1	88.9
Shang	VGG-19	91.2	90.5
Shang	ResNet-50	89.9	90.1
Zhou	SSA-CBAM-GNNs	**91.8**	**91.5**
Zhou	SVM	87.9	88.6
Zhou	CNN	87.9	88.6
Zhou	VGG-19	90.3	89.8
Zhou	ResNet-50	88.5	88.9
Han	SSA-CBAM-GNNs	**92.1**	**91.7**
Han	SVM	88.3	88.9
Han	CNN	89.9	88.5
Han	VGG-19	90.7	89.6
Han	ResNet-50	88.8	89.1
Tang	SSA-CBAM-GNNs	**92.3**	**91.9**
Tang	SVM	88.6	89.0
Tang	CNN	90.0	88.7
Tang	VGG-19	91.0	90.3
Tang	ResNet-50	89.2	89.5
Ming	SSA-CBAM-GNNs	**92.7**	**92.0**
Ming	SVM	88.9	89.3
Ming	CNN	90.5	89.1
Ming	VGG-19	91.5	90.8
Ming	ResNet-50	89.7	90.0
Overall	SSA-CBAM-GNNs	**92.2**	**91.7**
Overall	SVM	88.4	88.8
Overall	CNN	89.9	88.6
Overall	VGG-19	90.9	90.2
Overall	ResNet-50	89.2	89.5

The experiment aimed to evaluate the performance of various algorithms, including SSA-CBAM-GNNs, SVM, CNN, VGG-19, and ResNet-50, for the task of dynasty identification of ancient bronze drinking utensils. The dataset consisted of samples from five different dynasties: Shang, Zhou, Han, Tang, and Ming.

SSA-CBAM-GNNs consistently achieved the highest precision rates across all dynasties. For instance, in the Shang dynasty, SSA-CBAM-GNNs achieved a precision rate of 92.5, outperforming other algorithms. SVM, CNN, VGG-19, and ResNet-50 also demonstrated respectable precision rates, but they generally lagged behind SSA-CBAM-GNNs by a small margin. Similar to precision rates, SSA-CBAM-GNNs consistently exhibited the highest recall rates for each dynasty. For example, in the Han dynasty, SSA-CBAM-GNNs achieved a recall rate of 91.7, surpassing other algorithms. The other algorithms, including SVM, CNN, VGG-19, and ResNet-50, showed competitive recall rates but were outperformed by SSA-CBAM-GNNs. When considering the overall performance, SSA-CBAM-GNNs demonstrated the highest precision rate (92.2) and recall rate (91.7). It consistently outperformed the other algorithms across all dynasties.

The experimental results suggest that the SSA-CBAM-GNNs algorithm is highly effective for the identification and classification of ancient bronze drinking utensils by dynasty. It consistently achieved superior precision and recall rates compared to alternative algorithms.

## V. Conclusion

The success of SSA-CBAM-GNNs can be attributed to its unique combination of Sparrow Search Algorithm (SSA) for feature selection, Convolutional Block Attention Module (CBAM) for enhanced feature, and Graph Neural Networks (GNNs) for structured data processing. This combination allows SSA-CBAM-GNNs to capture the intricate cultural characteristics of bronze drinking utensils, including morphological and decorative features, and make accurate dynasty predictions.

While other algorithms such as SVM, CNN, VGG-19, and ResNet-50 performed reasonably well, SSA-CBAM-GNNs demonstrated a consistent edge in terms of accuracy. These findings indicate that the proposed SSA-CBAM-GNNs algorithm holds promise as a valuable tool for archaeologists and historians in the identification and classification of cultural relics, enriching our understanding of ancient societies based on their material culture.

Future research could involve the application of SSA-CBAM-GNNs to larger and more diverse datasets, potentially extending its use beyond bronze drinking utensils to other artifacts and relics. Moreover, fine-tuning the algorithm and exploring additional interpretability techniques could enhance its transparency and usefulness in cultural heritage identification and preservation.

## Supporting information

S1 File (DOCX)

## References

[1] Wu J.; Luo W.; Chen J.; Lin R.; Lyu Y. Design Ritual into Modern Product: A Case Study of Chinese Bronze Ware. Sustainability 2023, 15, 12747.

[2] Xu K.; Li Y.; Li Y.; Xu L.; Li R.; Dong Z. Masked Graph Neural Networks for Unsupervised Anomaly Detection in Multivariate Time Series. Sensors 2023, 23, 7552. doi: 10.3390/s23177552 37688008 PMC10490803

[3] Sun Y.; Wu I.-W.; Lin R. Transforming “Ritual Cultural Features” into “Modern Product Forms”: A Case Study of Ancient Chinese Ritual Vessels. Religions 2022, 13, 517.

[4] Li P.; Shi Z.; Ding Y.; Zhao L.; Ma Z.; Xiao H.; et al. Analysis of the Temporal and Spatial Characteristics of Material Cultural Heritage Driven by Big Data—Take Museum Relics as an Example. Information 2021, 12, 153.

[5] Tandon Y K, Bartholmai B J, Koo C W. Putting artificial intelligence (AI) on the spot: machine learning evaluation of pulmonary nodules. Journal of Thoracic Disease, 2020, 12(11): 6954–6965. doi: 10.21037/jtd-2019-cptn-03 33282401 PMC7711413

[6] Songa Y, et al. Component analysis and sub-classification of glass relics based on machine learning, Academic Journal of Computing & Information Science, 2023, 6(4): 49–56.

[7] Somrak Maja, Sašo Džeroski, and Žiga Kokalj. Learning to classify structures in ALS-derived visualizations of ancient Maya settlements with CNN. Remote Sensing.2020,14:2215.

[8] Hu X., Cao Y., Sun Y., Tang T. Railway automatic switch stationary contacts wear detection under few-shot occasions. IEEE Transactions on Intelligent Transportation Systems 2021, 23, 14893–14907.

[9] Awaji B.; Senan E.M.; Olayah F.; Alshari E. A., et al. Hybrid Techniques of Facial Feature Image Analysis for Early Detection of Autism Spectrum Disorder Based on Combined CNN Features. Diagnostics 2023, 13, 2948. doi: 10.3390/diagnostics13182948 37761315 PMC10527645

[10] Shafapourtehrany M.; Rezaie F.; Jun C.; Heggy E.; Bateni S.M.; Panahi M.; et al. Mapping Post-Earthquake Landslide Susceptibility Using U-Net, VGG-16, VGG-19, and Metaheuristic Algorithms. Remote Sens. 2023, 15, 4501.

[11] Bonhage A, Eltaher M, Raab T, Breuß M, Raab A, Schneider A. A modified Mask region-based convolutional neural network approach for the automated detection of archaeological sites on high-resolution light detection and ranging-derived digital elevation models in the North German Lowland. Archaeological Prospection. 2021,28:177–186.

[12] Argyrou A.; Agapiou A.; Papakonstantinou A.; Alexakis D.D. Comparison of Machine Learning Pixel-Based Classifiers for Detecting Archaeological Ceramics. Drones 2023, 7, 578.

[13] Amirah Hanani Jamil Fitri Yakub, et al. A Review on Deep Learning Application for Detection of Archaeological Structures, Journal of Advanced Research in Applied Sciences and Engineering Technology 2022,26(1): 7–14.

[14] Aricò M.; La Guardia M.; Lo Brutto M. 3D Data Integration for Web Fruition of Underground Archaeological Sites: A Web Navigation System for the Hypogeum of Crispia salvia (Marsala, Italy). Heritage 2023, 6, 5899–5918.

[15] Stanga C.; Banfi F.; Roascio S. Enhancing Building Archaeology: Drawing, UAV Photogrammetry and Scan-to-BIM-to-VR Process of Ancient Roman Ruins. Drones 2023, 7, 521.

[16] Penkova P.; Malcheva G.; Grozeva M.; Hristova T.; Ivanov G.; Alexandrov S.; et al. Laser-Induced Breakdown Spectroscopy and X-ray Fluorescence Analysis of Bronze Objects from the Late Bronze Age Baley Settlement, Bulgaria. Quantum Beam Sci. 2023, 7, 22.

[17] Crabu E.; Pes F.; Rodriguez G.; Tanda G. Ascertaining the Ideality of Photometric Stereo Datasets under Unknown Lighting. Algorithms 2023, 16, 375.

[18] Roelofs R, Shankar V, Recht B, et al. A meta-analysis of overfitting in machine learning. Advances in Neural Information Processing Systems, 2019, 32.

[19] Guidotti R, Monreale A, Giannotti F, et al. Factual and counterfactual explanations for black box decision making. IEEE Intelligent Systems, 2019, 34(6): 14–23.

[20] Zihni E, Madai VI, Livne M, et al. Opening the black box of artificial intelligence for clinical decision support: A study predicting stroke outcome. Plos one, 2020, 15(4): e0231166. doi: 10.1371/journal.pone.0231166 32251471 PMC7135268

[21] Borcherding A.; Morawetz M.; Pfrang S. Smarter Evolution: Enhancing Evolutionary Black Box Fuzzing with Adaptive Models. Sensors 2023, 23, 7864. doi: 10.3390/s23187864 37765921 PMC10537775

[22] Gabralla L.A.; Hussien A.M.; AlMohimeed A.; Saleh H.; Alsekait D.M.; El-Sappagh S.; et al. Automated Diagnosis for Colon Cancer Diseases Using Stacking Transformer Models and Explainable Artificial Intelligence. Diagnostics 2023, 13, 2939. doi: 10.3390/diagnostics13182939 37761306 PMC10529133

[23] Sun J.; Chen L.; Xia C.; Zhang D.; Huang R.; Qiu Z.; et al. CANARY: An Adversarial Robustness Evaluation Platform for Deep Learning Models on Image Classification. Electronics 2023, 12, 3665.

[24] Sun J, Li H, Fujita H, et al. Class-imbalanced dynamic financial distress prediction based on Adaboost-SVM ensemble combined with SMOTE and time weighting. Information Fusion, 2020, 54: 128–144.

[25] Gharehchopogh FS, Namazi M, Ebrahimi L, et al. Advances in sparrow search algorithm: a comprehensive survey. Archives of Computational Methods in Engineering, 2023, 30(1): 427–455. doi: 10.1007/s11831-022-09804-w 36034191 PMC9395821

[26] Yu Z.; Lei Y.; Shen F.; Zhou S.; Yuan Y. Research on Identification and Detection of Transmission Line Insulator Defects Based on a Lightweight YOLOv5 Network. Remote Sens. 2023, 15, 4552.

[27] Ye S.; Xu X.; Wang Y.; Fu T. Efficient Complex Aggregate Queries with Accuracy Guarantee Based on Execution Cost Model over Knowledge Graphs. Mathematics 2023, 11, 3908.

[28] Wang J., Zheng C., Yang X. and Yang L. Enhance Face: Adaptive Weighted SoftMax Loss for Deep Face Recognition. IEEE Signal Processing Letters.2022, 29:65–69.

[29] Xia K., Yin H., Qian P., Jiang Y. and Wang S. Liver Semantic Segmentation Algorithm Based on Improved Deep Adversarial Networks in Combination of Weighted Loss Function on Abdominal CT Images. IEEE Access, vol. 7, pp. 96349–96358, 2019.

[30] Zhang C, Ding S. A stochastic configuration network based on chaotic sparrow search algorithm. Knowledge-Based Systems, 2021, 220: 106924.

[31] Xu T., Wang Y., Zhang D., Zhao M. and Chen Y. Prediction on EMS of UAV’s Data Link Based on SSA-Optimized Dual-Channel CNN. IEEE Transactions on Electromagnetic Compatibility, vol. 64, no. 5, pp. 1346–1356, Oct. 2022.

[32] Sun G. et al. SpaSSA: Super pixel wise Adaptive SSA for Unsupervised Spatial–Spectral Feature Extraction in Hyperspectral Image. IEEE Transactions on Cybernetics, vol. 52, no. 7, pp. 6158–6169, July 2022. 34499610 10.1109/TCYB.2021.3104100

[33] Zhang X. et al. SSA-Net: Spatial Scale Attention Network for Image-Based Geo-Localization. in IEEE Geoscience and Remote Sensing Letters, vol. 19, pp. 1–5, 2022, Art no. 8022905.

[34] Xu X., Gao T., Wang Y. and Xuan X. Event temporal relation extraction with attention mechanism and graph neural network. Tsinghua Science and Technology, vol. 27, no. 1, pp. 79–90, Feb. 2022.

[35] Luo Z., Li J. and Zhu Y. A Deep Feature Fusion Network Based on Multiple Attention Mechanisms for Joint Iris-Periocular Biometric Recognition. IEEE Signal Processing Letters, vol. 28, pp. 1060–1064, 2021.

[36] Su E., Cai S., Xie L., Li H. and Schultz T. STAnet: A Spatiotemporal Attention Network for Decoding Auditory Spatial Attention From EEG. IEEE Transactions on Biomedical Engineering, vol. 69, no. 7, pp. 2233–2242, July 2022. 34982671 10.1109/TBME.2022.3140246

[37] Liang Y, Lin Y, Lu Q. Forecasting gold price using a novel hybrid model with ICEEMDAN and LSTM-CNN-CBAM. Expert Systems with Applications, 2022, 206: 117847.

[38] Wang Y, Zhang Z, Feng L, et al. A new attention-based CNN approach for crop mapping using time series Sentinel-2 images. Computers and electronics in agriculture, 2021, 184: 106090.

[39] Zhou J, Cui G, Hu S, et al. Graph neural networks: A review of methods and applications. AI open, 2020, 1: 57–81.

[40] Hu X., Cao Y., Tang T., Sun Y. Data-driven technology of fault diagnosis in railway point machines: Review and challenges. Transportation Safety and Environment 2022, 4, tdac036.

[41] Wu Z, Pan S, Chen F, et al. A comprehensive survey on graph neural networks. IEEE transactions on neural networks and learning systems, 2020, 32(1): 4–24. 10.1109/TNNLS.2020.2978386 32217482

[42] Scarselli F, Gori M, Tsoi AC, et al. The graph neural network model. IEEE transactions on neural networks, 2008, 20(1): 61–80. doi: 10.1109/TNN.2008.2005605 19068426

[43] Barthelemy P, Bertolotti J, Wiersma DS. A Lévy flight for light. Nature, 2008, 453(7194): 495–498. 18497819 10.1038/nature06948

