# Supplementary figures and images for: Research on bronze wine vessel classification using improved SSA-CBAM-GNNs
Source: PLoS One. 2024 Mar 21;19(3):e0295690. doi: 10.1371/journal.pone.0295690 (PMC10956876; doi:10.1371/journal.pone.0295690)

**Supporting Information files**

These are the minimal data set as a Supporting Information.


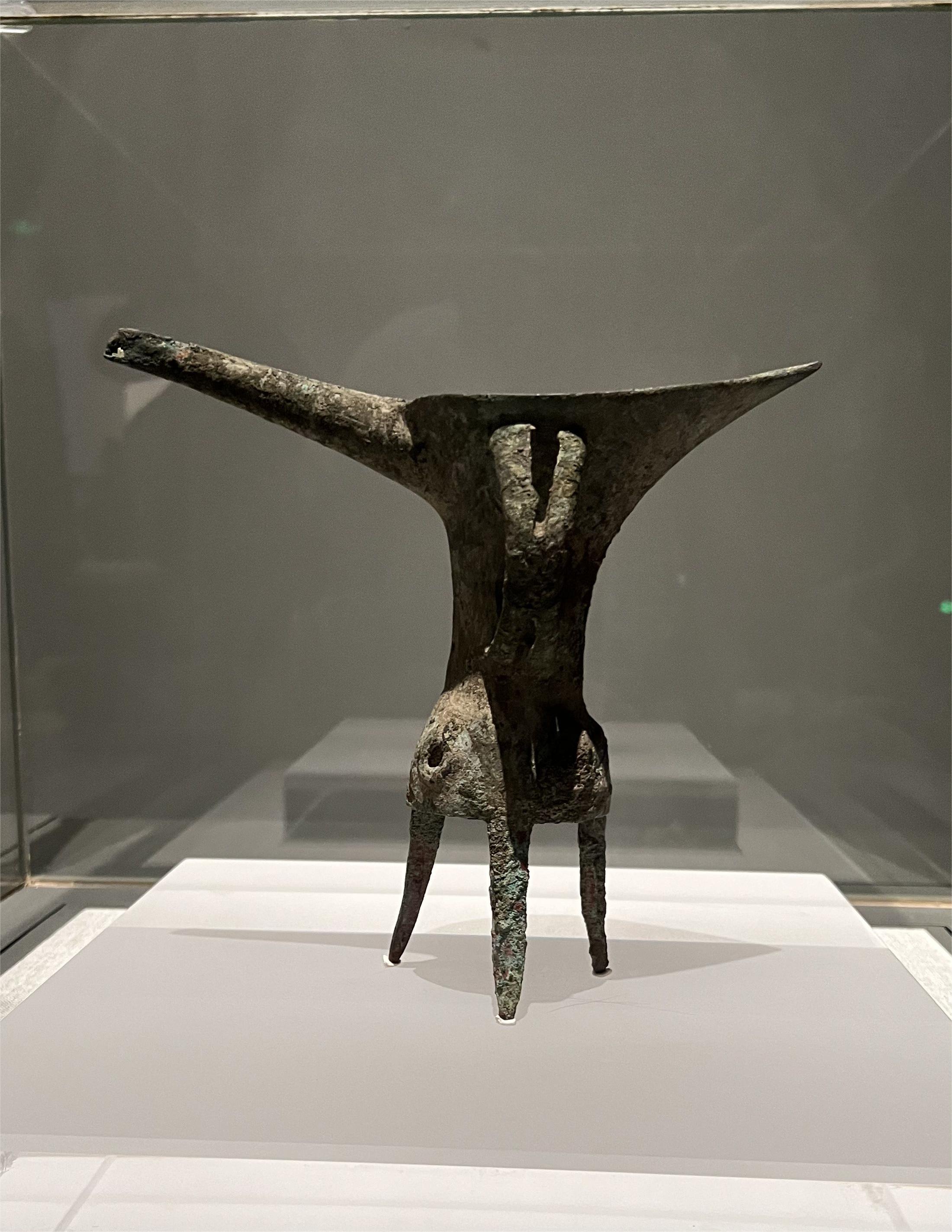

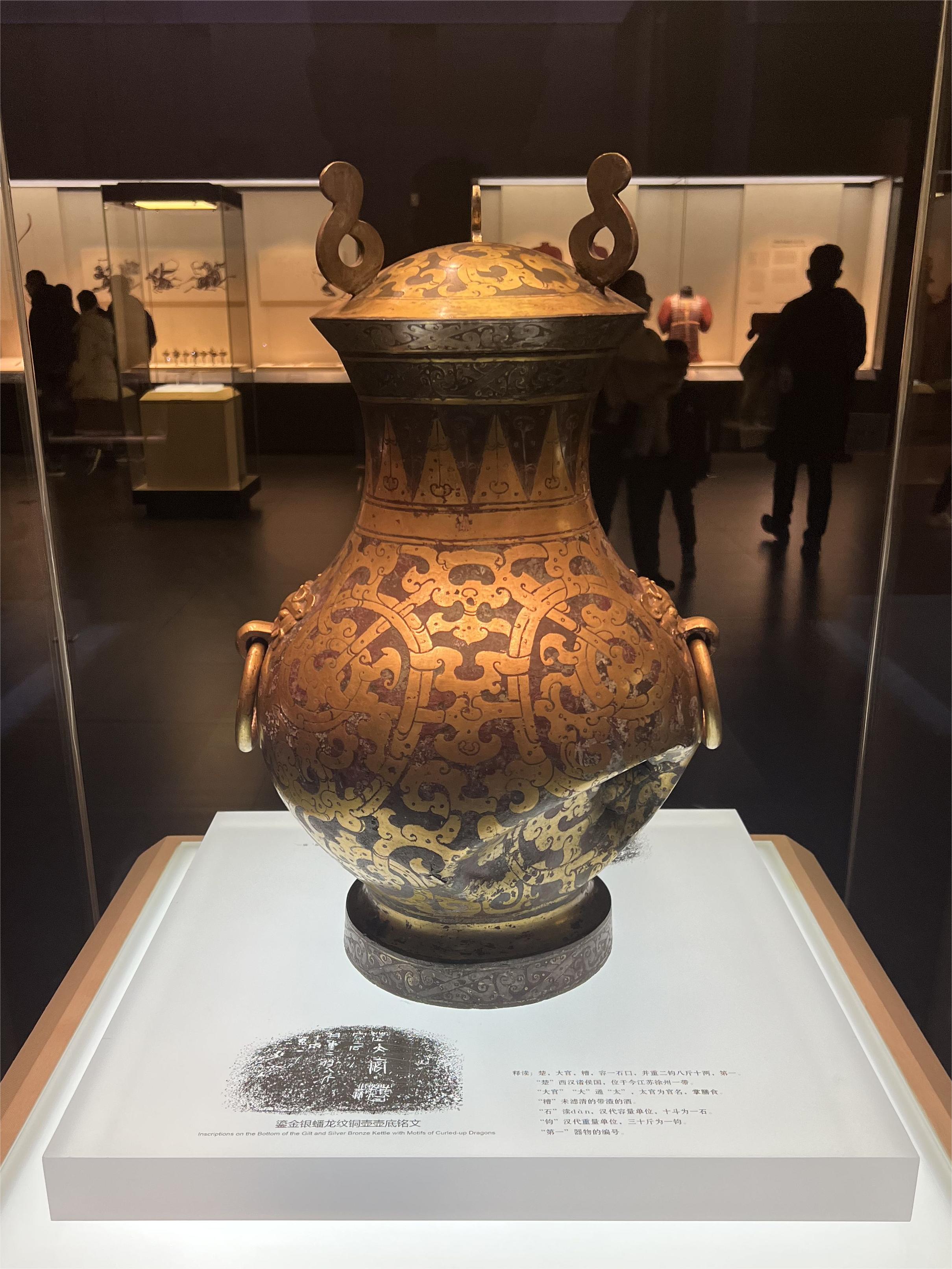

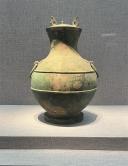

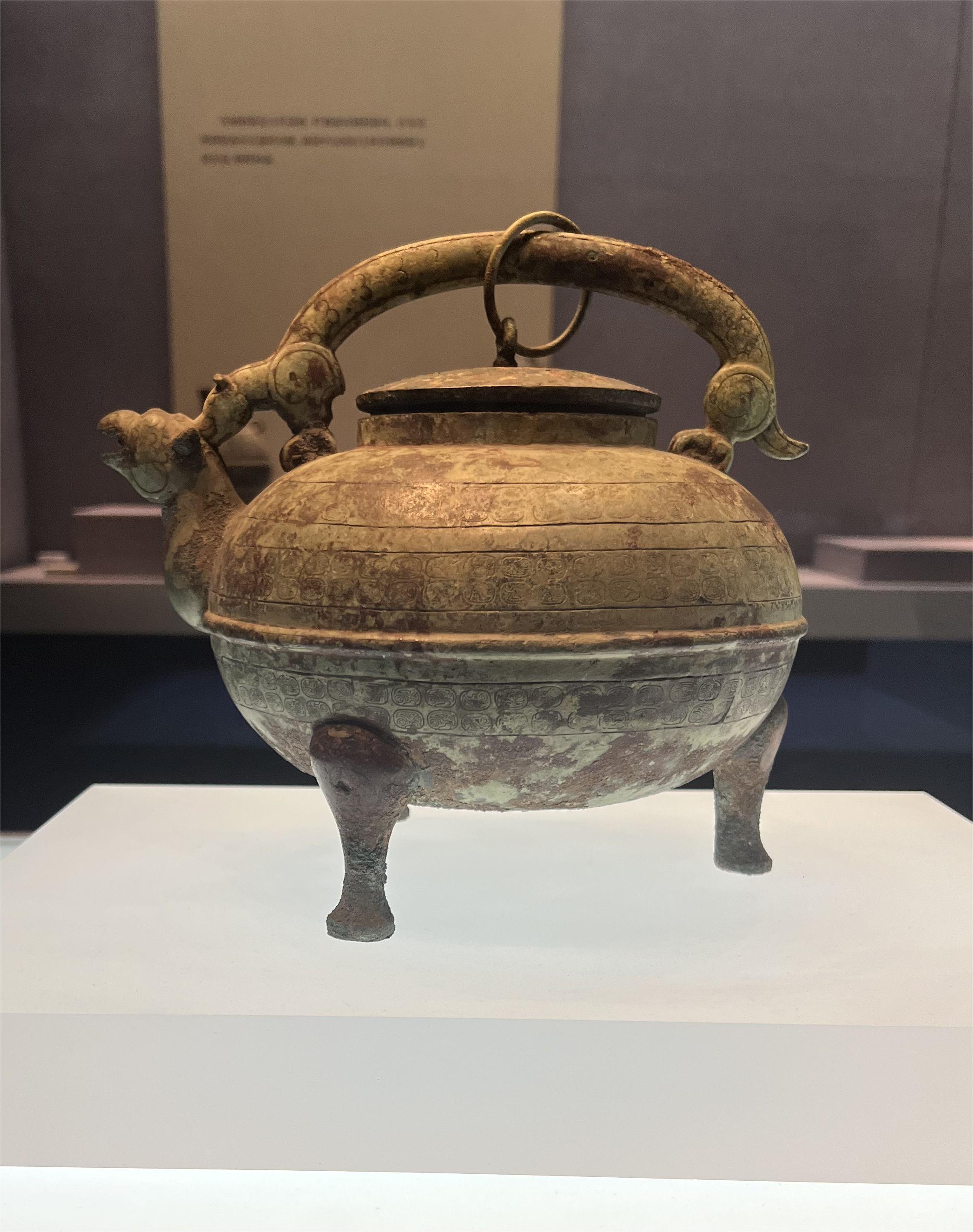


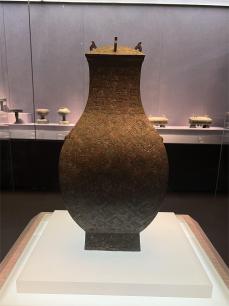

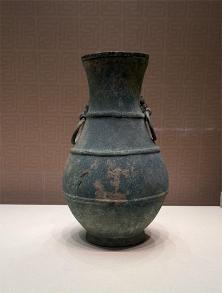

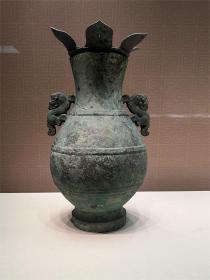

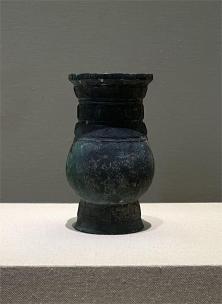

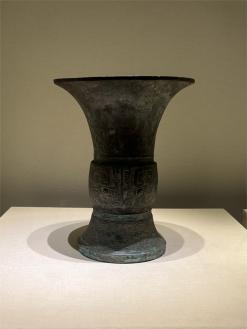

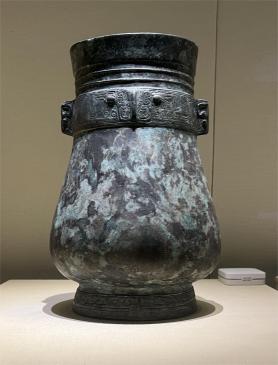

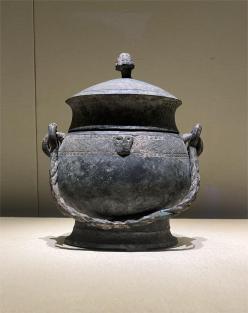

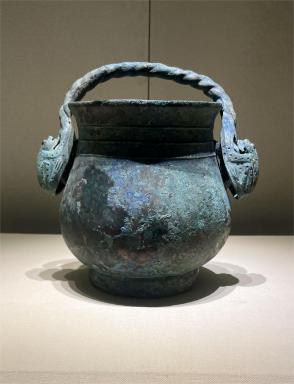

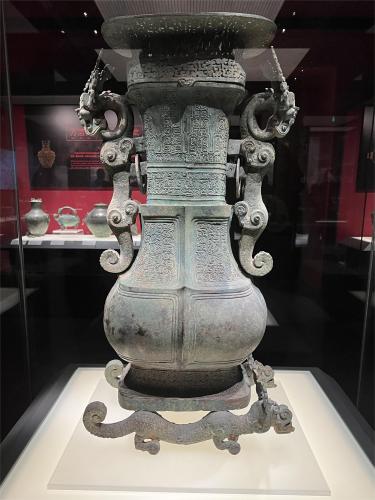

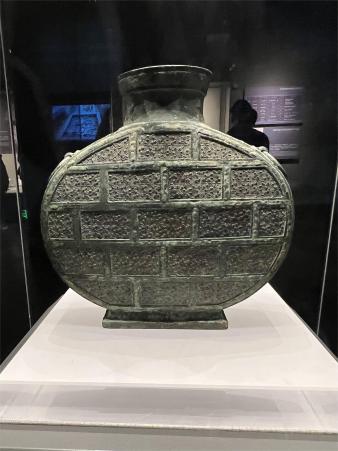

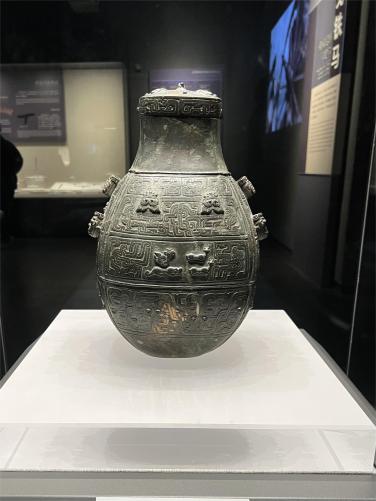

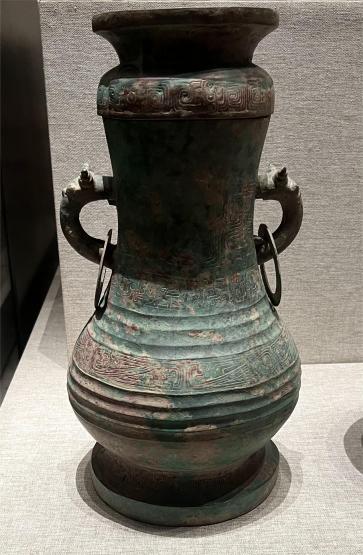

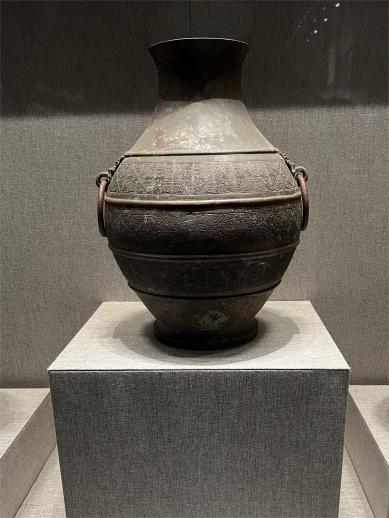

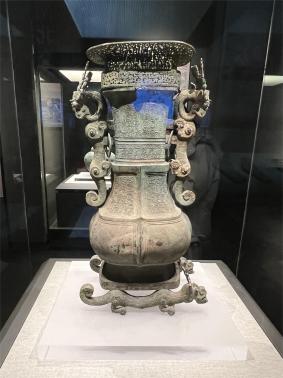

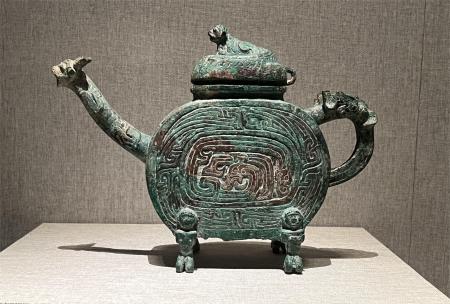

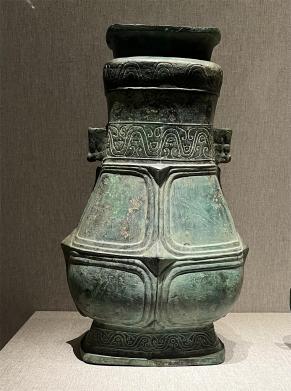

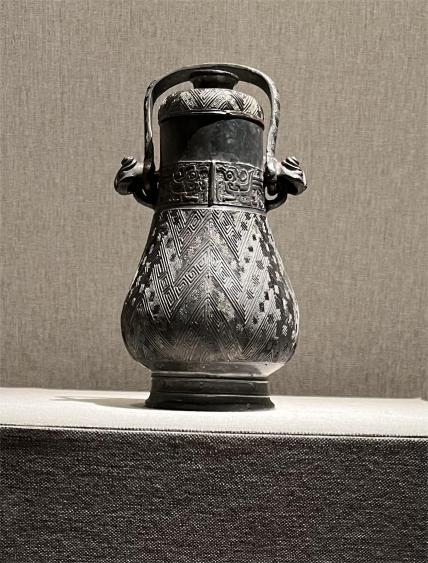

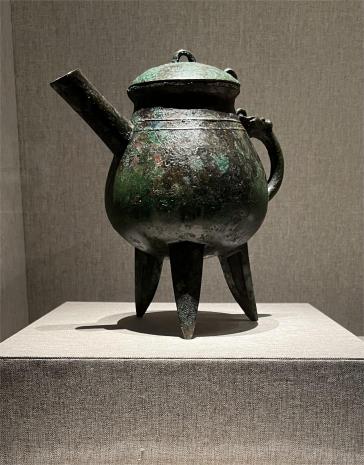

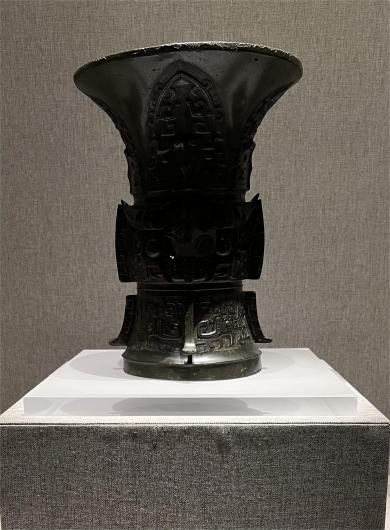

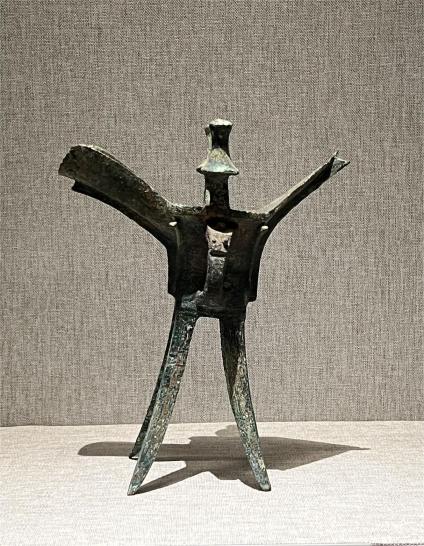

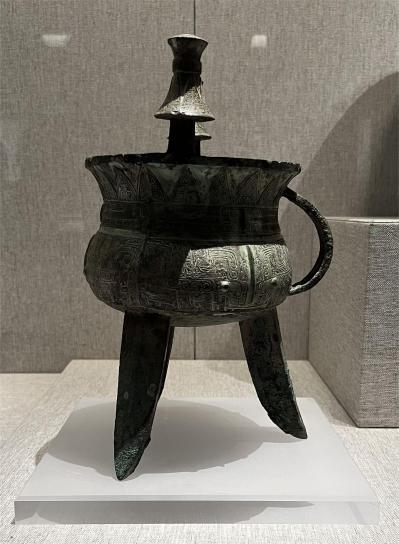

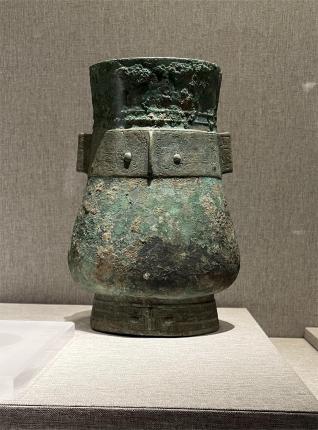

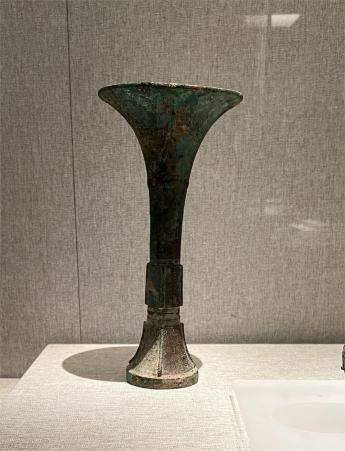

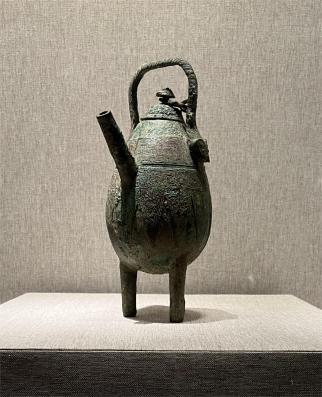

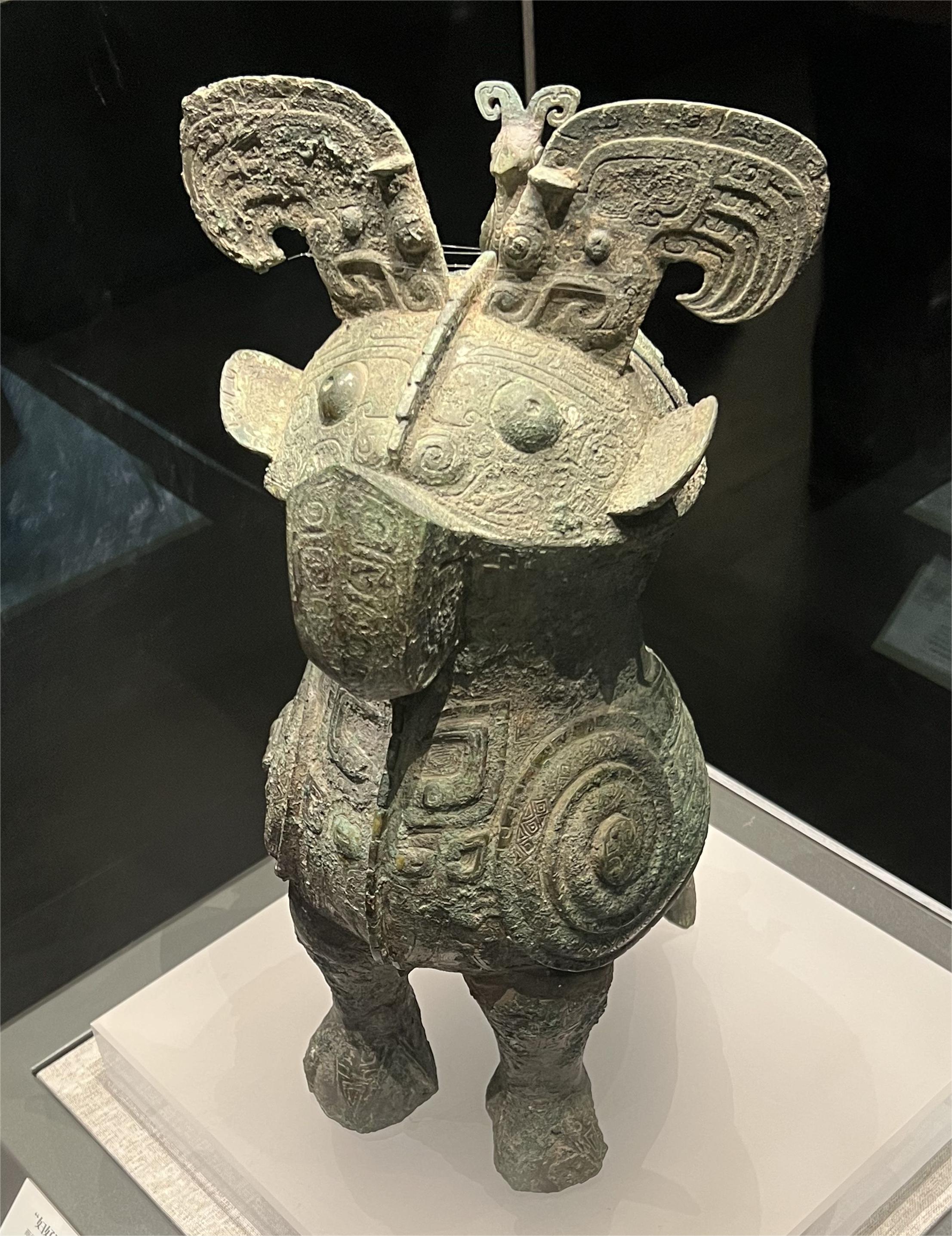

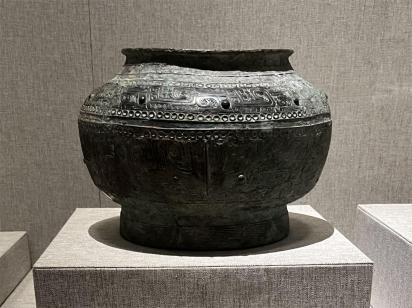

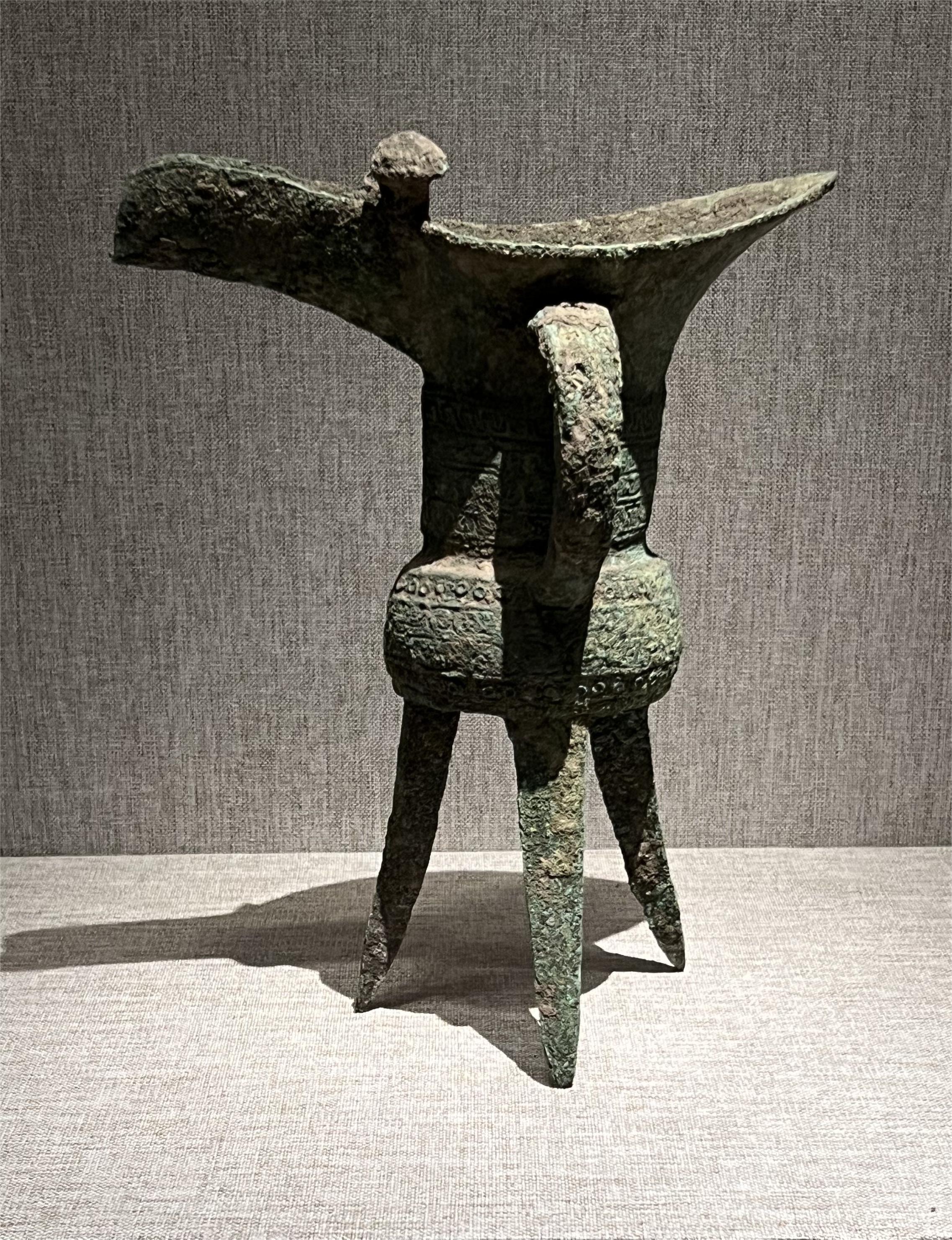

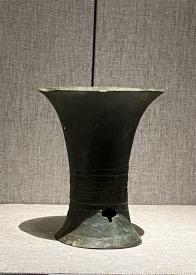

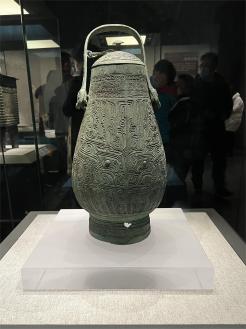

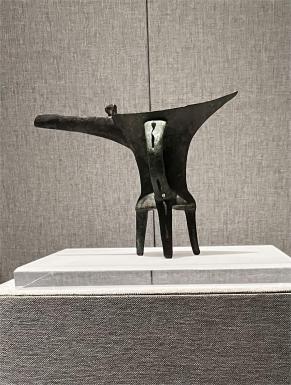


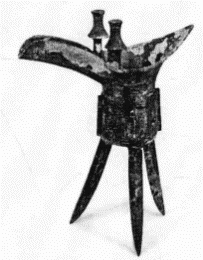

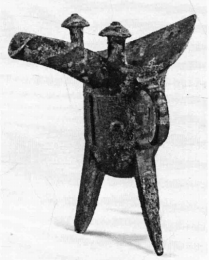

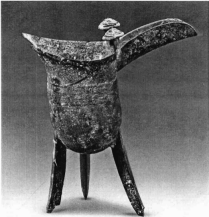

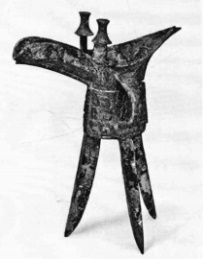

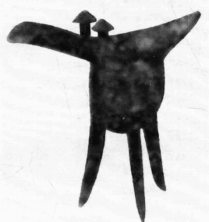

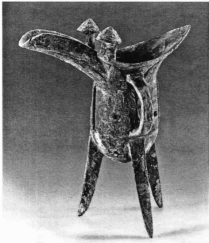


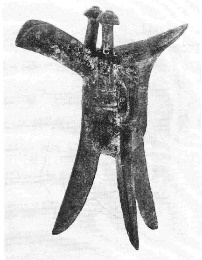

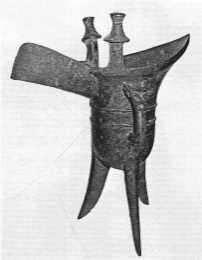

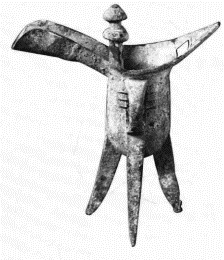

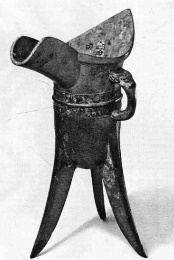

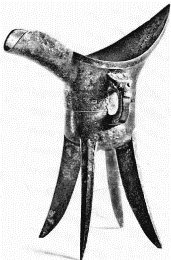

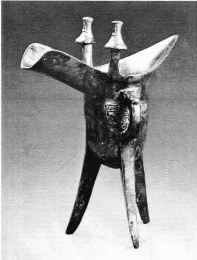

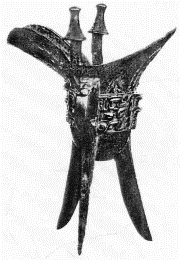


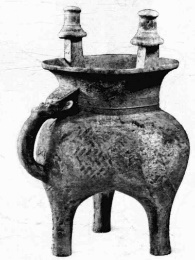

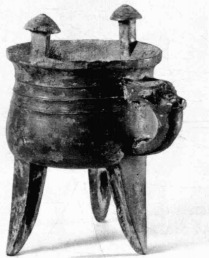

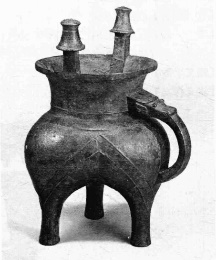

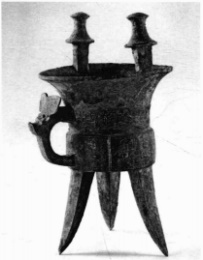

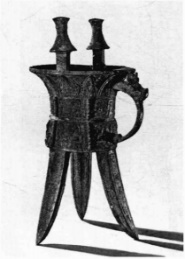

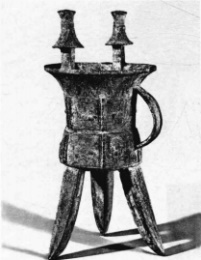

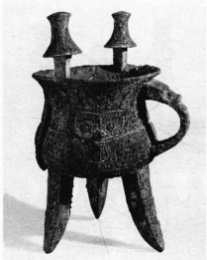


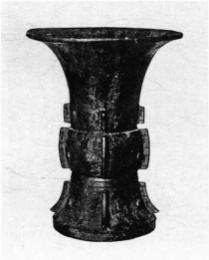

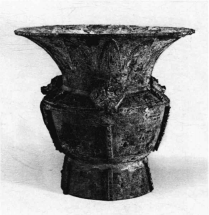

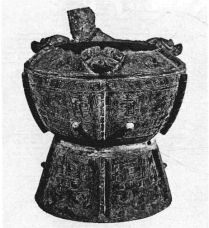

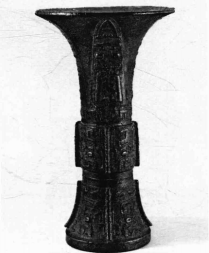

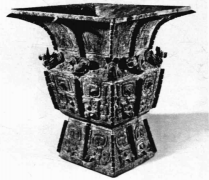

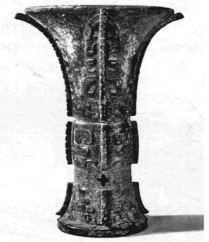


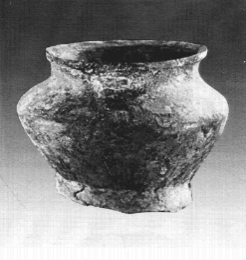

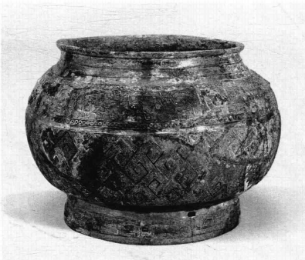

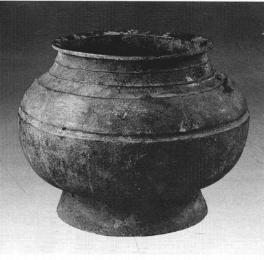

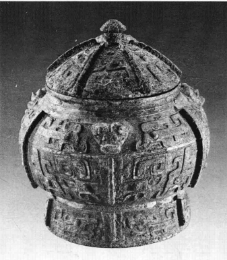

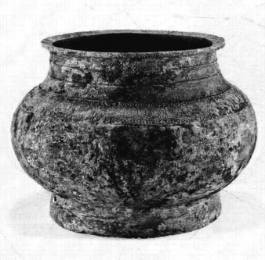

Supplement: S1 File — (DOCX) [file pone.0295690.s001.docx]
